# Temperature variability increases the onset risk of ischemic stroke: A 10-year study in Tianjin, China

**DOI:** 10.3389/fneur.2023.1155987

**Published:** 2023-04-14

**Authors:** Zhuangzhuang Chen, Peilin Liu, Xiaoshuang Xia, Lin Wang, Xin Li

**Affiliations:** ^1^Department of Neurology, The Second Hospital of Tianjin Medical University, Tianjin, China; ^2^Tianjin Interdisciplinary Innovation Centre for Health and Meteorology, Tianjin, China; ^3^Department of Geriatrics, The Second Hospital of Tianjin Medical University, Tianjin, China

**Keywords:** temperature variability, acute ischemic stroke, onset time, hypertension, seasons

## Abstract

**Background:**

Epidemiological evidence suggests a correlation between ambient temperature and ischemic stroke. However, evidence on the impact of daily temperature variability on the onset of ischemic stroke is lacking and limited.

**Objective:**

We aimed to investigate the short-term association between temperature variability and ischemic stroke occurrence in Tianjin.

**Methods:**

We performed a 10-year analysis of ischemic stroke patients hospitalized in two affiliated hospitals of Tianjin Medical University from 2011 to 2020. Daily meteorological data were collected from the Tianjin Meteorological Bureau. Temperature variability was calculated from the standard deviation (SD) of daily minimum and maximum temperatures over exposure days. A quasi-Poisson generalized linear regression combined with distributed lag non-linear model (DLNM) was used to estimate the effect of temperature variability on daily stroke onset, while controlling for daily mean temperature, relative humidity, long-term trend and seasonality, public holiday, and day of the week.

**Results:**

Temperature variability was positively associated with ischemic stroke. A 1°C increase in temperature variability at 0–1 days (TV_0–1_) was associated with a 4.1% (1.9–6.3%) increase of ischemic stroke onset. In a stratified analysis, men, people aged ≤65 years, and individuals with pre-existing hypertension, hyperlipidemia, hyperhomocysteinemia were more susceptible to temperature variability. Furthermore, the influence pattern of temperature variability on ischemic stroke was different in the cold season (November–April) and the warm season (May–October).

**Conclusion:**

Our findings suggested that short-term temperature variability exposure could increase the risk of ischemic stroke, which may provide new insights into the impact of climate change on health.

## 1. Introduction

Climate change has been considered potentially the greatest threat to human health in the 21st century, and more importantly, it will exacerbate global temperature changes, leading to increased frequency, duration and intensity of extreme weather events (such as heat waves, cold waves, and droughts). The impact of environmental temperature changes on people’s health is increasingly significant (1). Globally, stroke is the second leading cause of death and third leading cause of disability (2). In the past few decades, accumulating epidemiological studies have confirmed the association between stroke mortality and morbidity and ambient temperature (3, 4). Assessing the health risks of temperature changes is important for determining policy action on protecting public health. However, until now, the scientific evidence on the association between daily temperature variation and ischemic stroke is still insufficient.

Temperature variability (TV) is an important meteorological indicator reflecting climate change, such as rapid temperature fluctuations within a certain period (e.g., intra- and interday changes in temperature) (5, 6). It may also pose a significant threat to human health. Some previous studies have mentioned the association of temperature variation with ischemic stroke (IS). For example, rapid temperature drop and increased diurnal variation of temperatures were associated with increased IS risk (7, 8). However, these studies only considered the single intraday or interday changes of temperature, little evidence has been collected on the synthesized effects of temperature variation during interday and intraday periods. Until recent years, researchers have proposed a new method for assessing temperature variability, which well combines the intraday and interday variability of temperature, providing a theoretical basis for systematically assessing the association between temperature variability and ischemic stroke (5, 9).

Tianjin is located in the eastern part of the North China Plain, in the warm temperate semi-humid monsoon climate zone. It has four distinct climate characteristics, and the winter is controlled by Mongolia’s cold and high pressure, which is cold and dry. In summer, it is affected by the western side of the subtropical high in the northwest Pacific Ocean, with high temperature and high humidity. In the spring, it is dry and windy, and the cold and warm are changeable. Cold spells are also common in autumn. It can be seen that extreme weather such as cold spells and heat waves frequently occur in Tianjin (10). In addition, Tianjin is located in North China, where the prevalence of stroke is relatively high, and Tianjin can partially reflect the characteristics of stroke incidence in northern China (11). Therefore, the choice of this project to be carried out in Tianjin, where climate change is significant and the burden of stroke is heavy, is of great significance for reducing the stroke burden related to climate change in the region.

In this study, we designed a 10-year time-series study to investigate the short-term association between temperature variability and ischemic stroke in Tianjin, and attempted to identify susceptible populations for TV-induced ischemic stroke, so as to providing corresponding health guidance for stroke-susceptible individuals to protect them from the adverse effects of temperature variability.

## 2. Method

### 2.1. Data collection

The study population was patients admitted to the stroke units of the two affiliated Hospital of Tianjin Medical University due to acute ischemic stroke (ICD code I63) between January 1, 2011 and December 30, 2020 (*n* = 10,740). All patients underwent CT or MRI imaging after admission (within 24 h in most cases). The time of stroke onset was reported by patients and/or caregivers. 1,416 patients were excluded due to incomplete clinical data or the absence of new infarcts. Finally, 9,324 patients with acute cerebral infarction were included in the analysis. The patient’s past medical history data, such as: hypertension, diabetes (DM), hyperlipidemia, hyperhomocysteinemia (Hhcy), atrial fibrillation (AF), and coronary artery disease (CHD) were obtained from the hospital registration system. No protected health information was collected as data. Meteorological data during the corresponding period were obtained from the Tianjin Meteorological Bureau. Daily weather data includes daily minimum temperature (°C), daily mean temperature (°C), daily maximum temperature (°C), and daily mean relative humidity (%).

### 2.2. Exposure definition

As described in previous studies, we used a composite measure of intreday and intraday temperature changes to examine the effect of TV on ischemic stroke (5, 9). Temperature variability was calculated from the standard deviation (SD) of the daily minimum and maximum temperatures over the exposure days. For example, the temperature variability for the preceding 2 days’ exposure was calculated as follows: TV_0–1_ = SD (MinTemplag_0_, MaxTemplag_0_, MinTemplag_1_, MaxTemplag_1_). The TV for the preceding 3 days’ exposure was calculated by TV_0–2_ = SD (MinTemplag_0_, MaxTemplag_0_, MinTemplag_1_, MaxTemplag_1_, MinTemplag_2_, MaxTemplag_2_). This calculation method can well describe the intraday and interday temperature variation, as well as the lag effect of TV.

### 2.3. Data analysis

In the study period of 3,653 days, the maximum number of ischemic strokes in a single day was 10. Based on previous literature (5, 9, 12), a quasi-Poisson generalized linear regression model combined with distributed lag non-linear model (DLNM) was applied to estimate the relationship between TV and daily stroke onset, adjusting for daily mean temperature, relative humidity, long-term trend and seasonality, public holiday, and day of the week, as suggested by previous studies. First, the quasi-Poisson regression model was applied for estimating acute ischemic stroke onset associated with temperature variability exposure. We used a linear function for TV because previous studies had observed a broadly linear exposure-response curve between temperature variability and cardiovascular disease, and both large decreases and large increases in temperature between neighboring days increases the risk of health outcomes (6, 13). Then, a natural cubic spline was used to control long-term trends and seasonality. Additionally, relative humidity was controlled for using a natural cubic spline with 3 df. A categorical variable was used to control for the confounding effect of day of the week and public holiday. In order to eliminate the confounding effect of the daily mean temperature, we also include DLNM in the model to control the nonlinear and delayed effects of the daily mean temperature. Specifically, we used two natural cubic spline functions with 4 df, respectively, for daily mean temperature and lag over time up to 21 days to accommodate the nonlinear and lagged effects of ambient temperature. The selection of 21 days was based on the evidence that the effect of cold temperature was delayed and lasted for several weeks, while the effect of hot temperature was acute and generally presented within 1 week (4, 8, 14). Modeling was also repeated in the subgroup analysis (≤ 65 years vs. > 65 years; male vs. female; pre-exisiting history vs. without pre-exisiting history). Values with a two-tailed significance level less than 0.05 were considered statistically significant, and corresponding 95% confidence intervals (CIs) were used to describe effect estimates. All analysis was performed by R software (Version 4.0.3). All pictures were drawn by GraphPad Prism 8 software.

## 3. Results

### 3.1. Clinical data and weather conditions

The basic clinical characteristics of 9,324 patients with acute cerebral infarction who were hospitalized in the two affiliated hospitals of Tianjin Medical University from 2011 to 2020 were showed in Table 1. Overall, there were 60.03% male patients and 65.64% patients aged >65 years. Hypertension was the most common past history, accounting for 78.70%. Summary statistics of daily onset of ischemic stroke, weather conditions, and temperature variability were presented in Table 2. On average, we recorded 2 IS onset per day during the whole study period, with a range from 0 to 10. The annual-average daily temperature was 14.82°C, with a range from −12.87 to 34.26°C. The annual-average TV_0–1_ was 5.07°C, ranging from 0.92 to 12.4°C. The distributions of temperature variability were similar at different exposure days (TV_0–1_ to TV_0–7_) (see Supplementary Table 1). Exposure-response association curve between TV_0–1_ and daily stroke onset (see Supplementary Figure 1).

**Table 1:** Characteristics of acute ischemic stroke cases in this study.

Variable	Number	Proportion (%)
All	9,324	100.00%
Sex		
Male	5,597	60.03%
Female	3,727	39.97%
Age, years		
≤ 65	3,204	34.36%
>65	6,120	65.64%
Hypertension		
Yes	7,338	78.70%
No	1986	21.30%
Diabetes		
Yes	3,635	38.99%
No	5,689	61.01%
Hyperlipidemia		
Yes	3,788	40.63%
No	5,536	59.37%
Atrial fibrillation		
Yes	1,284	13.77%
No	8,040	86.23%
Hyperhomocysteinemia		
Yes	1791	19.21%
No	7,533	80.79%
Coronary heart disease		
Yes	4,527	48.55%
No	4,797	51.45%

**Table 2:** Summary statistics of daily onset for ischemic stroke, weather conditions, and temperature variability in Tianjin, 2011–2020.

Variables	Mean	Range
Ischemic stroke	2	0 to 10
Relative humidity (%)	50.16	7.63 to 95.21
Daily mean temperature (°C)	14.82	−12.87 to 34.26
Daily minimum temperature (°C)	10.83	−14.50 to 30.70
Daily maximum temperature (°C)	19.14	−11.60 to 40.10
Temperature variability		
TV_0–1_ (°C)	5.07	0.92 to 12.4
TV_0–2_ (°C)	4.96	1.13 to 11.53
TV_0–3_ (°C)	4.94	1.25 to 10.94
TV_0–4_ (°C)	4.95	1.67 to 10.66
TV_0–5_ (°C)	4.96	1.86 to 10.15
TV_0–6_ (°C)	4.97	2.11 to 9.63
TV_0–7_ (°C)	4.99	2.18 to 9.27

### 3.2. Temperature variability increases overall is risks

**Figure 1:**
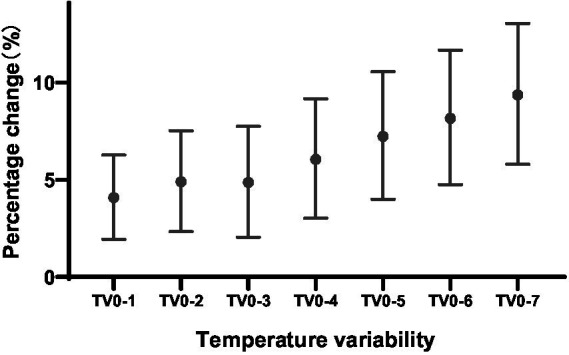
PC with 95% CI for ischemic stroke onset per 1°C increase in temperature variability at different exposure days. CI, confidence interval; PC, percentage change.

The percentage changes of ischemic stroke onset per 1°C increase in temperature variability with different exposure days were summarized in Figure 1. At 0–1, 0–2, 0–3, 0–4, 0–5, 0–6, and 0–7 days of exposure, the risk of ischemic stroke increased by 4.1% (95% CI: 1.9–6.3%, *P* < 0.001), 4.9% (95% CI: 2.3–7.5%, *P* < 0.001), 4.8% (95% CI: 2.1–7.8%, *P* < 0.001), 6.1% (95% CI: 3.0–9.2%, *P* < 0.001), 7.2% (95% CI: 4.0–10.6%, *P* < 0.001), 8.1% (95% CI: 4.7–11.7%, *P* < 0.001), and 9.4% (95% CI: 5.8–13.0%, *P* < 0.001), respectively.

### 3.3. Analysis results of different subgroups

**Figure 2:**
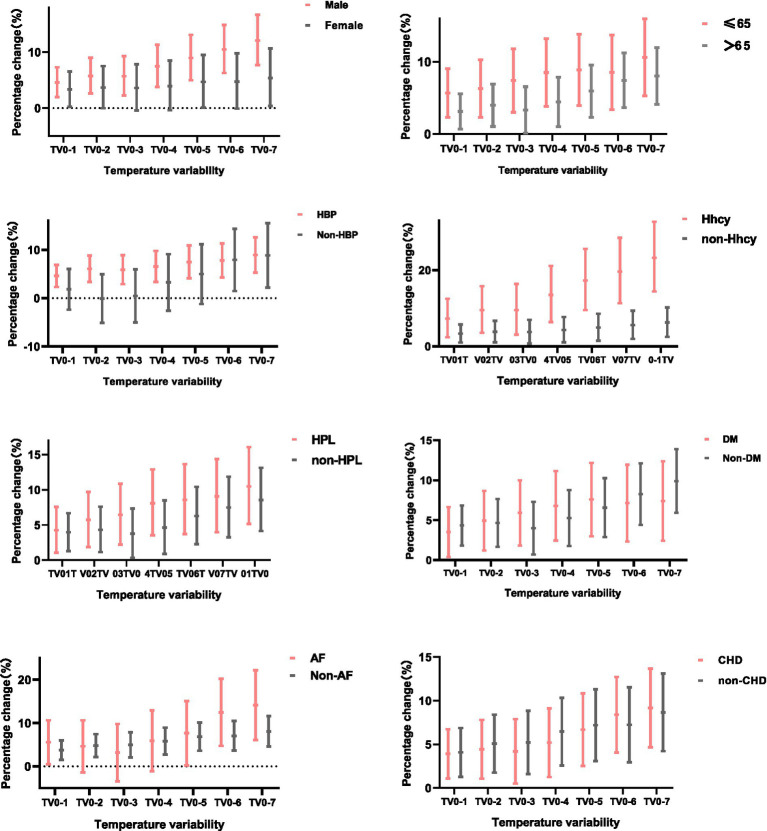
PC with 95% CI for ischemic stroke onset per 1°C increase in temperature variability at different exposure days, stratified by sex, age, and history of hypertension, DM, hyperlipidemia, Hhcy, CHD, and AF.

The results of the stratified analyses were demonstrated in Figure 2. The association between TV and ischemic stroke varied by gender. In female, a relatively weak association between TV and ischemic stroke was observed in TV_0–1_, TV_0–5_, and TV_0–7_. While in the male, exposure to TV was positively associated with ischemic stroke on all exposure days, with the largest estimates being observed at TV_0–7_. For a 1°C increase in TV_0–7_, we observed significant increases of 12.1% (7.7–16.7%) for IS onset in male. Moreover, we found that the effect of TV on stroke was significant for both people aged ≤65 years and the older, but higher effect estimates were observed in people aged <65 years. In patients aged ≤65 years, IS risk increased by 5.8% (2.3–9.5%) for every 1°C increase in temperature variability at 0–1 days of exposure. While, for patients aged >65 years, the risk of stroke increased by only 3.2% (0.7–5.7%) for every 1°C increase in TV_0–1_.

A stratified analysis of prior history found that patients with pre-existing hypertension were more likely to develop ischemic stroke due to temperature variability, especially on days 0–6 (8.1, 4.4–12.0%) and 0–7 (9.4, 5.4–13.4%) of exposure. No significance was observed in patients without hypertension on days 0–1 to 0–5. Additionally, we observed a stronger effect of TV on stroke incidence in patients with pre-existing hyperlipidemia and hyperhomocysteinemia at different exposure days than their counterparts. For patients with pre-existing AF, the largest estimates were observed at exposure 0–7 days, with every 1°C increase in temperature variability associated with 15.1% (6.3–24.8%) increase in IS onset, which was higher than that 8.4% (4.7–12.3%) in non-AF patients. Finally, a significant positive correlation between the TV and stroke onset was observed both in people with and without prior-DM or CHD.

### 3.4. Impact pattern of TV in cold season vs. warm season

**Figure 3:**
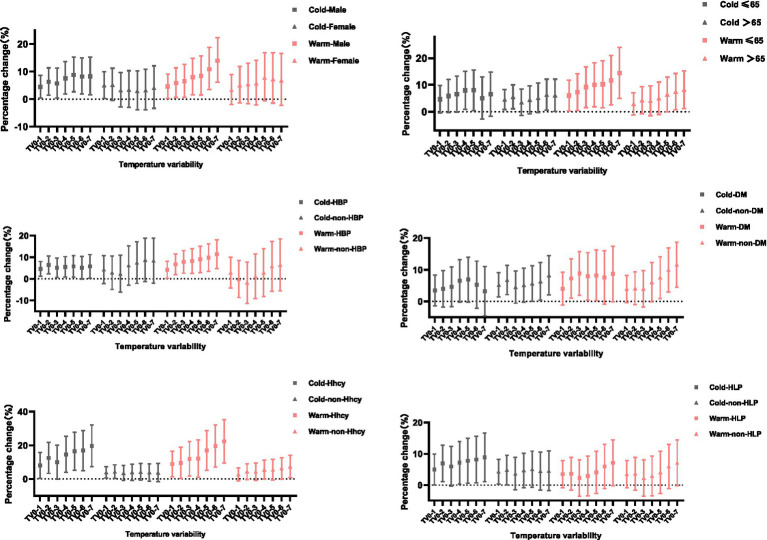
PC with 95% CI for ischemic stroke onset per 1°C increase in temperature variability at different exposure days, stratified by season and age, sex, and history.

When stratified by cold (November–April) and warm seasons (May–October), the influence of temperature variability was significant in both seasons, with stronger TV-stroke associations were found in warm season than in cold season. For every 1°C increase in TV_0–1_, the incidence of stroke in the cold and warm seasons increased by 6.5% (1.3–12.0%) and 11.0% (4.6–17.7%), respectively. Moreover, the influence patterns of TV in cold season and warm season on different subgroups were also quite different (Figure 3). It suggested that the men were more susceptible to temperature variability in warm season. For instance, at days 0–6, and 0–7 of TV exposure in the warm season, for every 1°C increase of TV, the IS risk for male increased by 10.8% (3.5–18.7%) and 13.9% (6.1–22.3%), respectively. While in the cold season, the risk increased by 8.2% (1.8–14.9%) and 8.2% (1.6–15.2%), respectively. Conversely, the female was more vulnerable to cold-season TV, and the effect reached statistical significance on days 0–1 in the cold season, while not on any exposure days in the warm season. Furthermore, compared with the cold season, individuals aged ≤65 years were more affected by temperature variability in the warm season, while people aged >65 years were significantly affected by TV_0–1_ and TV_0–2_ in the cold season, as well as TV_0–6_ and TV_0–7_ days in both cold and warm season.

The influence patterns of TV on the patients with pre-existing history (e.g., hypertension, DM, hyperlipidemia, Hhcy) in the cold season and the warm season were also different. For hypertensive patients, positive associations between TV and ischemic stroke were observed on 0–1 to 0–5 and 0–7 exposure days in the cold season, as well as all the exposure days in the warm season. Noteworthy, for non-hypertensive patients, the effect of TV on stroke onset did not reach statistical significance on both seasons. Likewise, for patients with a history of hyperlipidemia, the effect of TV in the cold season was higher than that in the warm season. Moreover, we observed a positive association between TV and IS onset on days 0–2 to 0–5 in the warm season for DM patients, but not in any exposure days in the cold season. Finally, for patients with previous Hhcy, TV on different exposure days in the warm and cold season had a significant impact on the incidence of stroke, except for TV_0–3_ in the cold season.

## 4. Discussion

This article considers both intraday and daytime temperature changes in an attempt to fully assess the acute effects of temperature changes on ischemic stroke. Previous studies have focused on the effect of temperature change on disease mortality. For example, pooled estimates for 12 counties in Hubei Province, China, showed a 1.72% (0.69–2.76%) increase in cardiovascular mortality for each 1°C increase in TV_0–7_ (15). A recent study of 31 major cities in China showed that a 1°C increase in TV_0–7_ was associated with a 0.60% (0.25–0.94%) increase in cardiovascular mortality (9). To date, there was only a handful of literature to assess the effect of temperature variability on cardiovascular morbidity, and the timing of patient admission rather than onset was often used, which may impair the accuracy of the effect of TV on stroke morbidity. This present study was the first to assess the effect of TV on stroke onset by matching temperature variability to precise stroke onset timing. In line with previous regional and national studies, which demonstrated that high diurnal temperature range (DTR) was associated with hospital admission for ischemic stroke, and for every 1°C increase in DTR, the risk of stroke increased by 9% (2–16%) (16, 17), we also found a positive correlation between temperature variability and stroke morbidity, with a 4.1% (1.9–6.3%) increase in ischemic stroke onset for every 1°C increase in TV_0–1_.

In the present study, we observed that men are more susceptible to temperature variability than women for ischemic stroke, which was in agreement with previous studies (18). Evidence suggested that men may undertake more physical work and outdoor exercise than women, which may make them more vulnerable to weather changes (19). Additionally, differences in physiological conditions and hormone levels between the two genders may also be one of the potential influencing factors (20). However, some other studies found that females were more sensitive to temperature changes (21–23). Further researches for the reasons underlying these differences are required. In a stratified analysis of age, our study found that both people aged ≤65 and those aged >65 years were susceptible to temperature variability, with people aged ≤65 years being more affected. The influence of temperature variability in younger populations may be related to more opportunities for occupational exposure, while decreased thermoregulation and higher prevalence of chronic diseases also partly explain the increased vulnerability of older people to temperature changes (22–24). Furthermore, older individuals always have limited information on the weather forecast and the impending large TV, which may further increase their vulnerabilities to the TV exposure. At present, there is still some heterogeneity in the research conclusions of different regions, which may be related to the climate and geographical region of each region (22, 25, 26). In addition, characteristics of specific cities, such as sociodemographics, chronic disease burden or lifestyle, and population susceptibility, may also be responsible for heterogeneity.

This study was the first to analyze the effect of temperature variability on stroke incidence in individuals with different comorbidities. Our results found that compared with their counterparts, individuals with previous hypertension, hyperlipidemia and hyperhomocysteinemia were more susceptible to temperature variability. Additionally, a significant positive correlation between the TV and stroke onset was observed both in people with and without prior-DM or CHD. This is consistent with previous epidemiological evidence, patients with hypertension (27, 28), hyperglycemia and hyperlipidemia are susceptible to extreme temperature and temperature fluctuations (29), and biochemical indicators such as blood pressure, blood glucose, and blood lipids are poorly controlled and abnormally increased, which may induce the occurrence of cardio-cerebrovascular diseases under temperature fluctuations (30–33). Until now, the underlying molecular biological mechanisms of the harmful effects of temperature changes on ischemic stroke have yet to be explored. Humans maintain core body temperature within a narrow range (about 37°C) by exchanging heat with the surrounding environment. However, when confronted with sudden changes in temperature within a short time period, the human thermoregulatory system might not respond efficiently to promptly balance the body temperature with ambient temperature (34). Several mechanisms suggest that unstable temperature may alter heart rate, blood cholesterol levels, blood pressure, plasma fibrinogen concentrations, platelet activity, and inflammatory markers in the bloodstream, which may exacerbate the risk of thrombosis (10, 27, 35, 36).

Finally, the impact patterns were different in cold season (November–April) and warm season (May–October) after stratifications. We found that the influence of temperature variability on stroke in both seasons was significant, with stronger TV-morbidity associations were found in warm season than in cold season. For example, for every 1°C increase in TV_0–1_ in the warm season, the incidence of stroke increased by 11.0% (4.6–17.7%) and by 6.5% (1.3–12.0%) in the cold season, respectively. This result was consistent with a national time series study from 184 cities in China, which reported that the estimates of TV for all health conditions were higher in warm season than in cool season (12). In a previous study in Hubei Province, higher estimates of TV-related stroke mortality were also observed during the warm season, with 2.62% (1.08, 4.18%) and 1.37% (0.03, 2.74%) in the warm and cold seasons, respectively (15). In a subgroup analysis of 65 years of age in the cold and warm seasons, our results showed that people aged ≤65 years were susceptible to TV_0–7_ in the warm season but not in the cold season, while people aged >65 years were affected by TV_0–7_ in the cold and warm seasons, which is also consistent with previous studies (15).This findings suggest that we should formulate more accurate protection strategies based on the influence of temperature variability on people of different ages. Furthermore, patients with previous hypertension and hyperlipidemia were more susceptible to cold season temperature variability, while patients with previous DM were more susceptible to warm season temperature variability. Therefore, it is necessary to optimize the prevention and treatment strategies of TV-related stroke for different vulnerable populations. For example, it may be effective to provide some health guidance and temperature warning for patients with hypertension and hyperlipidemia in the cold season and DM patients in the warm season.

The strength of this study was that we analyzed clinical data over a multi-year span and provided comprehensive estimates after adjusting for several confounding factors. Moreover, the estimates were based on stroke onset time rather than the admission time, which provided a more accurate prediction and avoided unnecessary bias. Furthermore, for the first time, we performed a stratified analysis based on common comorbidities and identified several vulnerable people with TV-related strokes, which is important for future public health policy development. Meanwhile, some limitations in the present study should also be noted. Although the two affiliated hospitals of Tianjin Medical University are among the largest in Tianjin, covering nearly half of Tianjin’s stroke patients. However, since the study was conducted primarily in urban areas, the validity of the estimates for suburban and rural areas around Tianjin may be weakened. In addition, due to the unavailability of data, this study failed to adjust for air pollution. But, previous investigations have shown that the health effects of temperature variation are generally robust and even independent of air pollution (12, 37). Moreover, the daily ambient temperatures were collected at fixed sites rather than at the individual level, which may lead to some measurement errors. Finally, we used outdoor ambient temperature to evaluate the association of TV and ischemic stroke, and did not evaluate indoor temperature exposure, which may cause some bias.

## 5. Conclusion

Short-term exposure to increased temperature variability is associated with an increased risk of ischemic stroke, especially in men, age ≤ 65 years, and individuals with pre-existing hypertension, hyperlipidemia and hyperhomocysteinemia. It is recommended to establish an early warning system to report the upcoming large temperature variations in a timely and effective manner, so as to provide corresponding protection measures for stroke-susceptible individuals.

## Data availability statement

The raw data supporting the conclusions of this article will be made available by the authors, without undue reservation.

## Ethics statement

The studies involving human participants were reviewed and approved by Institutional Ethics Committee of the Second Hospital of Tianjin Medical University (No. KY2020K142). Written informed consent for participation was not required for this study in accordance with the national legislation and the institutional requirements.

## Author contributions

XL contributed to the study design. ZC, PL, XX, and LW contributed to statistical analysis and manuscript draft. All authors helped to perform the analysis and to revise the manuscript with constructive discussions and contributed to the article and approved the submitted version.

## Funding

This work was supported by the Tianjin Municipal Science and Technology Bureau Project (21JCZDJC01230), the Tianjin Key Medical Discipline (Specialty) Construction Project (TJYXZDXK-065B), and the National Natural Science Foundation of China (42275197).

## Conflict of interest

The authors declare that the research was conducted in the absence of any commercial or financial relationships that could be construed as a potential conflict of interest.

## Publisher’s note

All claims expressed in this article are solely those of the authors and do not necessarily represent those of their affiliated organizations, or those of the publisher, the editors and the reviewers. Any product that may be evaluated in this article, or claim that may be made by its manufacturer, is not guaranteed or endorsed by the publisher.
