# Supplementary material for: Temperature variability increases the onset risk of ischemic stroke: A 10-year study in Tianjin, China
Source: Front Neurol. 2023 Apr 14;14:1155987. doi: 10.3389/fneur.2023.1155987 (PMC10140412; doi:10.3389/fneur.2023.1155987)
Supplement: Supplementary file 1 [file Table_1.docx]

**S1 Table.** The distribution of daily temperature variability (TV) at different exposure days in Tianjin, 2011–2020.

| Variable | Mean ± SD | Minimum | 25th | 50th | 75th | Maximum |
| --- | --- | --- | --- | --- | --- | --- |
| TV_0–1_ (°C) | 5.07 ± 1.54 | .92 | 4.00 | 4.96 | 6.03 | 12.40 |
| TV_0–2_ (°C) | 4.96 ± 1.33 | 1.13 | 4.03 | 4.85 | 5.80 | 11.53 |
| TV_0–3_ (°C) | 4.94 ± 1.23 | 1.25 | 4.06 | 4.84 | 5.73 | 10.94 |
| TV_0–4_ (°C) | 4.95 ± 1.16 | 1.67 | 4.12 | 4.82 | 5.71 | 10.66 |
| TV_0–5_ (°C) | 4.96 ± 1.12 | 1.86 | 4.17 | 4.84 | 5.71 | 10.15 |
| TV_0–6_ (°C) | 4.97 ± 1.09 | 2.11 | 4.18 | 4.85 | 5.69 | 9.63 |
| TV_0–7_ (°C) | 4.99 ± 1.06 | 2.18 | 4.21 | 4.85 | 5.69 | 9.27 |
