# Supplementary material for: Temperature variability increases the onset risk of ischemic stroke: A 10-year study in Tianjin, China
Source: Front Neurol. 2023 Apr 14;14:1155987. doi: 10.3389/fneur.2023.1155987 (PMC10140412; doi:10.3389/fneur.2023.1155987)
Supplement: Supplementary file 2 [file Data_Sheet_1.docx]

**S1. Fig** Exposure-response association curve between TV0–1 and daily stroke onset for Tianjin in China, 2011–2020. TV0–1, temperature variability at 0–1 days.
